# Behind the screen: Unraveling the role of perceived closeness difference in shaping viewers’ engagement behaviors

**DOI:** 10.1371/journal.pone.0347751

**Published:** 2026-07-06

**Authors:** Runhua Huang, Huichao Guo

**Affiliations:** 1 Business Technology Innovation and Development Research Center (Hangzhou), Zhejiang Business College, Hangzhou, China; 2 Wuhan University of Technology, Wuhan, China; Para Federal University, BRAZIL

## Abstract

Viewer engagement is central to the live-streaming economy. While existing literature primarily examines the dyadic relationship between a viewer and a streamer, it largely overlooks the social context: viewers simultaneously witness the streamer’s real-time interactions with peers. Drawing on Leader-Member Exchange (LMX) differentiation and Social Comparison Theory, we introduce *Perceived Closeness Difference* (PCD), defined as the cognitive gap between a viewer’s perceived closeness to the streamer and their perception of a peer’s closeness. Across six experimental studies (*N* = 1,980), we examine how PCD is associated with engagement behaviors. Study 1 shows that witnessing a streamer favor a peer (Negative PCD) is associated with lower engagement, whereas witnessing peer rejection (Positive PCD) is associated with higher engagement, partly mediated by participants’ subjectively measured PCD scores. Studies 2 and 3 identify boundary conditions, indicating that these effects are stronger in emotional (vs. utility) contexts and among more emotionally oriented viewers. Study 4 shows that the pattern varies with the peer’s group identity (in-group vs. out-group). Study 5 indicates that PCD is more strongly related to public/social engagement (e.g., commenting) than to private/financial engagement. Finally, Study 6 links PCD to long-term loyalty via perceived streamer trustworthiness. Together, these findings extend one-and-a-half-sided relationship perspectives and offer implications for community management in interactive media.

## Introduction

The rise of live streaming represents a paradigm shift in digital interaction. Unlike static media, live streaming offers a synchronized, immersive environment where content creation and consumption happen simultaneously [1]. This industry has evolved into a dominant force in the global digital economy, driven by the audience’s desire for real-time engagement [2]. However, for streamers, the challenge has shifted from merely attracting eyeballs to fostering sustained psychological bonds. While financial gifting and subscriptions are critical metrics, they are merely the downstream behavioral outcomes of complex upstream psychological processes [3,4].

To understand these processes, scholars have predominantly relied on the framework of Parasocial Interaction (PSI) [5,6]. This extensive body of work posits that viewers form intimate, illusory bonds with media figures, driven by the streamer’s attractiveness, expertise, and responsiveness [7–9]. While valuable, this traditional perspective has an important limitation, which we refer to as *dyadic myopia*. It implicitly models the viewer-streamer relationship as an isolated, one-to-one channel, ignoring the broader social reality of the platform.

A live stream is inherently a *shared* social space. A viewer does not interact with the streamer in a vacuum; they interact within a visible, scrolling feed of peers [10]. Consequently, viewers are not only observers of the streamer, but also observers of the *streamer’s interactions with others*. Recent media theory describes this as a one-and-a-half-sided relationship [11], yet empirical models quantifying how witnessed third-party interactions impact the focal viewer remain scarce. Social Comparison Theory suggests that, in high-visibility social environments, individuals evaluate their standing relative to others [12–14]. We argue that the streamer’s differential treatment of viewers may function as a salient signal of social standing, with consequences for viewers’ responses.

To operationalize this dynamic, we bridge media psychology with organizational behavior, drawing a novel parallel between streamers and organizational leaders. Much like leaders in virtual teams [15,16], streamers manage a hierarchy of relationships. The theory of Leader-Member Exchange (LMX) differentiation posits that employees are sensitive not just to their own treatment, but to how their treatment compares to that of their peers (Relative LMX) [17,18]. When a leader displays favoritism, it violates norms of fairness and demotivates the wider team [19].

Building on this cross-disciplinary logic, we introduce the construct of **Perceived Closeness Difference (PCD)**. We define PCD as a specific type of social comparison: the cognitive gap between a viewer’s perceived closeness to the streamer and their perception of a peer’s closeness ($\text{PCD}={\text{Closeness}}_{\text{Own}}-{\text{Closeness}}_{\text{Other}}$). Unlike general envy or jealousy, PCD is a comparative cognitive assessment of relational status. We propose that PCD functions as a relational comparison cue. A *Negative PCD* (witnessing a peer being favored) may trigger upward social comparison, posing a threat to the viewer’s identity and status within the community [20] and reducing engagement. Conversely, a *Positive PCD* (witnessing a peer being rejected) may trigger downward social comparison, reinforcing the viewer’s sense of status and distinctiveness [21], thereby increasing engagement.

In this paper, we present six experimental studies to examine the PCD construct and its consequences. We aim to (1) test the effect of PCD on engagement behaviors beyond personal preference, (2) identify boundary conditions across content types (emotional vs. utility) and viewer dispositions, (3) examine the moderating role of social identity (in-group vs. out-group dynamics), and (4) assess downstream associations with streamer trustworthiness and loyalty. This research offers a broader view of live streaming by incorporating community dynamics alongside viewer-streamer intimacy.

## Literature review

To ground the construct of Perceived Closeness Difference (PCD) and its theoretical mechanisms, we review three interrelated streams of literature: (1) Parasocial Interaction in live streaming, (2) Social Comparison Theory in digital environments, and (3) Leader-Member Exchange (LMX) differentiation. We then synthesize these perspectives to develop our formal hypotheses.

### Parasocial interaction and live streaming engagement

The concept of parasocial interaction (PSI) was first introduced by Horton and Wohl [5] to describe the illusory sense of intimacy that audiences develop with media performers. Rubin et al. [22] subsequently operationalized PSI, demonstrating that such relationships are driven by perceived proximity, attentiveness, and the regularity of media exposure. Giles [6] consolidated these findings in a comprehensive review, establishing PSI as a robust predictor of media consumption behavior.

With the advent of live streaming, PSI has taken on new dimensions. Unlike traditional broadcast media, live streaming affords real-time, bidirectional communication between streamers and viewers [11]. Recent empirical work has confirmed that PSI remains a central driver of engagement in this new context. Lee et al. [7] demonstrated cross-cultural stability of PSI effects on viewing intentions, while Yen et al. [8] integrated PSI within the Elaboration Likelihood Model to predict purchase behavior. Huang [9] further showed that PSI, combined with social presence, drives impulsive purchasing in live commerce.

McLaughlin and Wohn [3] advanced the field by identifying community-level predictors of PSI, suggesting that viewers’ sense of belonging to a streamer’s community matters beyond the dyadic bond. Kowert and Daniel [11] formalized this intuition, proposing a “one-and-a-half-sided” relationship model in which the interactive affordances of live streaming create a qualitatively different parasocial experience. However, despite this theoretical advance, empirical models that quantify how *witnessed third-party interactions* within the community affect the focal viewer’s engagement remain scarce. This gap motivates the present research.

### Social comparison theory in digital environments

Festinger’s [12] Social Comparison Theory posits that individuals evaluate their own abilities and opinions by comparing themselves to others, particularly when objective standards are unavailable. Buunk and Gibbons [23] extended this framework by demonstrating individual differences in social comparison orientation, showing that some people are dispositionally more prone to engage in comparative evaluation.

In digital environments, social comparison processes have been shown to have profound consequences. Vogel et al. [13] demonstrated that exposure to idealized social media profiles triggers upward social comparison, resulting in lower self-esteem. Le Blanc-Brillon et al. [24] confirmed similar effects on young adults’ mental health through Instagram use. Conversely, Rüther et al. [25] showed that downward social comparison with influencers can enhance self-esteem under certain conditions. Liu et al. [26] identified social media envy as a mediator between social comparison and consumption intentions.

Despite this rich literature, existing social comparison research in digital contexts has focused almost exclusively on *static* platforms (e.g., Facebook profiles, Instagram posts). The *real-time*, *public*, and *interactive* nature of live streaming creates a fundamentally different social comparison environment. In a live stream, viewers witness the streamer’s differential treatment of peers *as it happens*, making the comparison immediate, vivid, and difficult to dismiss. Our construct of PCD captures this specific form of real-time social comparison.

### Leader-member exchange (LMX) differentiation

Leader-Member Exchange theory, as reviewed by Martin et al. [27], posits that leaders develop differentiated relationships with individual followers, ranging from high-quality exchanges (characterized by trust, mutual respect, and obligation) to low-quality, transactional relationships. A meta-analysis by these authors confirmed the robust link between LMX quality and individual performance outcomes.

Crucially, recent work has shifted attention from absolute LMX quality to *relative* LMX—how an individual’s exchange quality compares to that of peers. Lee et al. [17] demonstrated that employees engage in social comparison of their LMX standing, and that perceiving one’s LMX as lower than a peer’s triggers felt obligation and psychological entitlement. Martin et al. [18] provided conceptual clarification of LMX differentiation, linking it to justice perceptions and team-level outcomes. Choi et al. [19] showed that perceived LMX differentiation harms team performance by generating conflict and perceptions of injustice. Liden et al. [28] established that differentiation effects depend on task interdependence, suggesting that context moderates the impact of relative standing.

We draw a novel parallel between leaders and streamers. Like leaders in virtual teams [15,16], streamers manage a hierarchy of relationships in a digital environment where interactions are visible to all members. When a streamer publicly “favors” one viewer over another, this is functionally equivalent to a leader displaying LMX differentiation, with the critical distinction that in live streaming, the differentiation is performed *publicly* in front of the entire community.

### Hypotheses development

Synthesizing the three theoretical pillars above, we propose the following hypotheses:

Based on the PSI and social comparison frameworks, we predict that: **H1**: Witnessing a streamer’s differential treatment of a peer viewer will significantly influence the focal viewer’s engagement behavior (positively when the peer is rejected; negatively when the peer is favored), even after controlling for the viewer’s baseline preferences.

To establish the mechanism, we propose: **H2**: Perceived Closeness Difference (PCD) mediates the effect of witnessed peer interactions on engagement.

Drawing on the distinction between emotional and utilitarian consumption [29,30], we predict: **H3**: The effect of PCD on engagement is stronger in emotionally-engaged streaming contexts than in utility-focused contexts.

Based on individual differences in social comparison orientation [23], we predict: **H4**: The indirect effect of PCD on engagement is stronger for viewers with a higher emotional (vs. rational) disposition.

Grounded in Social Identity Theory [20], we predict: **H5a**: Negative PCD (favoritism) will produce a stronger negative effect on engagement when the favored peer belongs to the viewer’s in-group (“betrayal” effect). **H5b**: Positive PCD (rejection) will produce a stronger positive effect on engagement when the rejected peer belongs to an out-group (intergroup distinctiveness effect).

Considering the social-evaluative nature of PCD, we predict: **H6**: PCD will have a stronger effect on public/social engagement behaviors (e.g., commenting, sharing) than on private/financial engagement behaviors (e.g., tipping, purchasing).

Finally, linking PCD to downstream relational outcomes, we predict: **H7**: PCD affects viewer loyalty indirectly through its impact on perceived streamer trustworthiness.

## Materials and methods

### Overview and ethics statement

All studies reported in this manuscript were conducted in accordance with the Declaration of Helsinki. The research protocol was approved by the Institutional Review Board (IRB) of Zhejiang Business College (Ref: ZJBC-2025-Exp003). All participants provided informed consent electronically prior to participation. Data collection took place between September 2024 and November 2024. To ensure statistical power, we conducted an *a priori* power analysis using G*Power 3.1. Based on a medium effect size (*f* = 0.25), an alpha level of 0.05, and a power of 0.80 for a one-way ANOVA, a minimum sample size of 159 was required. We over-recruited in all studies to account for potential attention-check failures.

### Study 1: The main effect of perceived closeness difference (PCD)

#### Participants and design.

A total of 210 undergraduate students were recruited from Zhejiang Business College in exchange for course credit (61% Female, $M_{age}=21.4,SD=1.8$). Participants were randomly assigned to a one-factor, three-level between-subjects design (PCD Condition: Negative vs. Positive vs. Control).

#### Procedure.

**Fig 1:**
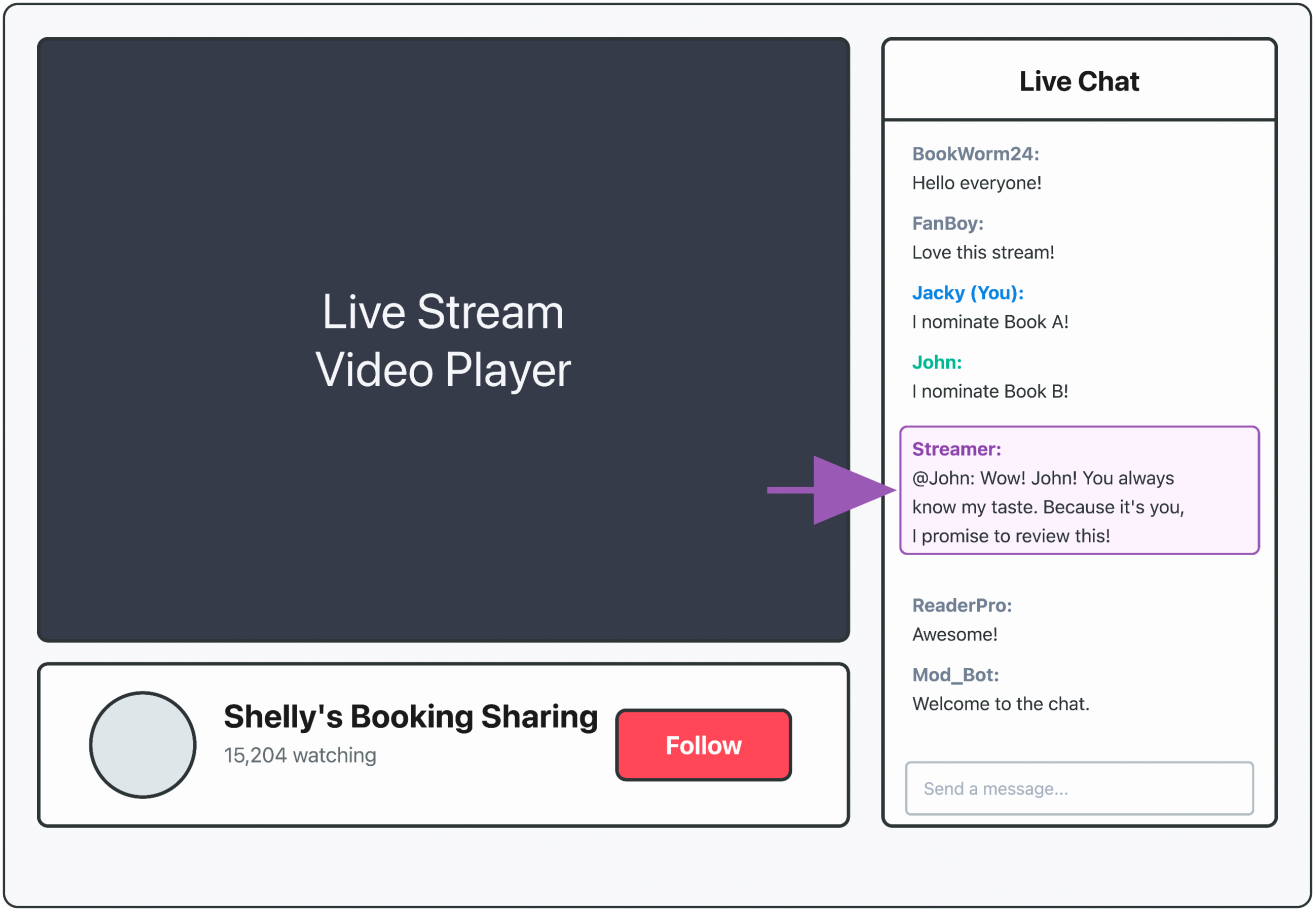
The real-time simulation interface used to present the experimental manipulation. Participants witnessed a pre-scripted interaction (indicated by purple arrows) designed to manipulate Perceived Closeness Difference (PCD). *Note: The interface depicted here is a custom schematic for illustrative purposes to comply with open-access CC BY 4.0 guidelines. The actual screenshots of the simulation interface used during the experiments are publicly available in the study’s GitHub repository.*

We developed a high-fidelity, web-based live streaming simulator using Python and HTML5. Upon logging in, participants were assigned a pseudonym (e.g., “Jacky”) and entered a chat room interface mimicking popular platforms (e.g., TikTok, Twitch). The visual layout of this simulated environment, including the real-time chat stream and video player, is depicted in Fig 1.

The procedure consisted of three phases:

1. **Immersion Phase (4 min):** Participants watched a pre-recorded, 4-minute book-sharing session featuring a professional streamer.
2. **Baseline Measurement:** To control for individual preferences, participants were asked to gift virtual coins (range: 0–10) to the streamer. This score served as a covariate in subsequent analyses.
3. **PCD Manipulation:** The streamer invited viewers to nominate a book for the next session. The participant (Jacky) and a simulated peer confederate (John) both submitted nominations. The AI-driven backend then delivered the experimental manipulation via the streamer’s verbal response and on-screen chat text.

#### Experimental manipulation.

The streamer acknowledged the participant’s gift with standard gratitude in all conditions. The manipulation focused on the interaction with the peer (John):

- **Control Condition (Equal Treatment):** The streamer treated John with the same neutral politeness as the participant (e.g., Thanks for the suggestion, John. I will consider it.).
- **Negative PCD Condition (Peer Favored):** The streamer exhibited excessive enthusiasm towards John (e.g., Wow! John! You always know my taste. Because it’s you, John, I promise to review this book next time!). This condition was designed to elicit ${\text{Closeness}}_{\text{Own}}<{\text{Closeness}}_{\text{Other}}$.
- **Positive PCD Condition (Peer Rejected):** The streamer publicly dismissed John (e.g., Oh, John again... honestly, I really dislike that author. I have to say no to you, John. Never gonna happen.). This condition was designed to elicit ${\text{Closeness}}_{\text{Own}}>{\text{Closeness}}_{\text{Other}}$.

#### Measures.

*Perceived Closeness Difference (PCD):* We measured PCD using a two-item closeness assessment comparing the focal viewer’s perceived relationship with that of the peer viewer.

*Engagement Index:* Engagement was measured using a 7-item composite scale adapted from [2] and [3]. Items included I would subscribe to this channel, I would share this stream, and I would send paid gifts. The scale showed high internal consistency (α=0.92). All items were rated on a 1–10 scale.

### Study 2: Moderation by streamer type

#### Participants and Design.

Participants (*N* = 420) were recruited from two universities and randomly assigned to a 3 (PCD: Negative, Positive, Control) × 2 (Streamer Type: Emotionally-Engaged vs. Utility-Focused) between-subjects design (37% Female, $M_{age}=21.8,SD=1.9$).

#### Procedure.

The procedure mirrored Study 1, with the addition of a *Streamer Type* manipulation.

- **Emotionally-Engaged Context:** The live stream featured an attractive singer performing emotional ballads. The streamer used language emphasizing connection, feelings, and soul [29].
- **Utility-Focused Context:** The live stream featured a professional technology reviewer analyzing the specifications of a new laptop. The streamer used technical jargon and focused on objective metrics [30].

A manipulation check asked participants to rate the primary focus of the stream (1 = Purely Information, 7 = Purely Entertainment), confirming the distinction ($M_{Util}=2.3$ vs. $M_{Emo}=5.8,p<\mathrm{.001}$).

### Study 3: Moderation by viewer disposition

#### Participants and procedure.

We recruited 300 participants online (58% Female, $M_{age}=22.1,SD=2.0$). Before the experiment, participants completed the *Affective Orientation Scale* (AOS) [31] to measure their dispositional tendency to rely on emotions versus reason. Participants were then randomized into the three PCD conditions using the Study 1 paradigm (dance performance context). The dependent variable was the Engagement Index (α=0.92).

### Study 4: Moderation by peer social identity

#### Participants and design.

This study employed a 2 (PCD: Negative vs. Positive) × 2 (Peer Identity: In-Group vs. Out-Group) between-subjects design (*N* = 440; 60% Female, $M_{age}=21.6,SD=1.9$).

#### Manipulation of peer identity.

We manipulated social identity using a Fan Badge paradigm adapted from [32].

- **In-Group Condition:** The interface displayed a Gold Badge next to the participant’s name. The peer John also displayed a Gold Badge. The streamer remarked, I see John is a Gold Member, just like you, [Participant Name].
- **Out-Group Condition:** The participant held a Gold Badge, but John displayed a Rival Clan badge. The streamer noted, I see John is visiting from the [Rival] fan group.

Following this identity prime, the streamer delivered the PCD manipulation scripts (Favoritism or Rejection) as described in Study 1.

### Study 5: Differential impact on engagement types

#### Participants and measures.

Participants (*N* = 330; 59% Female, $M_{age}=21.9,SD=1.8$) were assigned to the three PCD conditions. We distinguished between two types of engagement behaviors based on factor analysis:

1. **Public/Social Engagement:** Three items measuring visible actions: commenting, sharing to social media, and recommending to friends (α=0.87).
2. **Private/Financial Engagement:** Three items measuring high-cost or private actions: purchasing advertised products, paid subscriptions, and anonymous tipping (α=0.76).

### Study 6: Downstream consequences on trust and loyalty

#### Participants and measures.

Participants (*N* = 280; 62% Female, $M_{age}=22.0,SD=1.7$) were assigned to the PCD conditions. In addition to engagement, we measured:

- **Perceived Streamer Trustworthiness:** Measured using a 3-item scale adapted from [4] (e.g., This streamer treats viewers fairly, I trust this streamer’s integrity; α=0.93).
- **Viewer Loyalty:** Measured using 3 items assessing long-term intent (e.g., I will continue watching this streamer in the future; α=0.87).

## Results

### Preliminary analysis

Prior to hypothesis testing, we inspected the data for normality and outliers. Skewness and kurtosis values for all dependent variables were within the acceptable range (±1.5). Randomization checks confirmed no significant differences in demographic variables (age, gender) across conditions (*p* > .15).

To provide an overview of the research program, Table 1 summarizes the design, manipulations, and key findings across the six studies.

**Table 1:** Summary of experimental designs and key findings (Studies 1-6).

Study	Objective	Design	Key Manipulations (IVs)	Key DVs	Key Finding
1	Establish the main effect of PCD and mediation.	3-group (PCD: Neg, Pos, Control)	Witnessed peer interaction (positive, negative, neutral).	Engagement Index, Measured PCD.	PCD mediates the effect of witnessed interactions on engagement (H2).
2	Test moderation by streamer type.	3 (PCD) x 2 (Streamer Type)	PCD manipulation; Streamer content (Emotional vs. Utility).	Engagement Index.	PCD effect is amplified for emotional streamers and attenuated for utility streamers (H3).
3	Test moderation by viewer disposition.	3 (PCD) x Continuous (Disposition)	PCD manipulation; Viewer Disposition (measured *a priori*).	Engagement Index, Measured PCD.	PCD effect is amplified for emotionally-driven viewers (H4).
4	Test moderation by peer’s social identity.	2 (PCD: Neg, Pos) x 2 (Peer Identity)	PCD manipulation; Peer Identity (In-group vs. Out-group).	Engagement Index.	Favoritism of an in-group peer is most damaging (H5a); rejection of an out-group peer is most beneficial (H5b).
5	Test differential impact on engagement type.	3-group (PCD: Neg, Pos, Control)	PCD manipulation.	Public/Social Engagement Index; Private/Financial Engagement Index.	PCD effect is stronger for public/social DVs and not significant for private/financial DVs (H6).
6	Test downstream consequences on loyalty.	3-group (PCD: Neg, Pos, Control)	PCD manipulation.	Perceived Streamer Trustworthiness, Viewer Loyalty.	The effect of PCD on loyalty is mediated by perceived streamer trustworthiness (H7).

### Study 1: The main effect of PCD and mediation analysis

Study 1 examined whether witnessed peer interactions influence focal viewer engagement beyond personal preference (H1) and whether PCD mediates this relationship (H2). An Analysis of Covariance (ANCOVA) was conducted on the Engagement Index, with PCD Condition as the independent variable and *baseline preference* as a covariate. The covariate was significant, $F(1,206)=6.24,p=\mathrm{.013},\eta_{p}^{2}=\mathrm{.03}$. Crucially, after controlling for preference, there was a significant main effect of PCD Condition, $F(2,206)=31.92,p<\mathrm{.001},\eta_{p}^{2}=\mathrm{.24}$.

Post-hoc pairwise comparisons (Bonferroni-corrected) revealed distinct patterns (see Table 2). Participants in the *Positive PCD* (Peer Rejected) condition reported significantly higher engagement ($M_{adj}=5.75,SD=1.07$) compared to the *Control* condition ($M_{adj}=4.94,SD=1.09;p<\mathrm{.001},95\%CI[0.46,1.18]$). Conversely, participants in the *Negative PCD* (Peer Favored) condition reported significantly lower engagement ($M_{adj}=4.28,SD=1.15$) than the *Control* group (p<.001,95%CI[−1.02,−0.28]). These findings support H1.

**Table 2:** Study 1: Adjusted means, standard deviations, and 95% confidence intervals.

PCD Condition	*M*_*adj*_	*SD*	95% CI for Mean	
			Lower	Upper
Positive PCD (Peer Rejected)	5.75	1.07	5.49	6.01
Control (Equal Treatment)	4.94	1.09	4.68	5.20
Negative PCD (Peer Favored)	4.28	1.15	4.03	4.54

*Note.* Means are adjusted for baseline preference (covariate); SDs are observed within-condition standard deviations.

**Mediation Analysis.** To test the mediating role of PCD (H2), we utilized Hayes’ PROCESS macro (Model 4) with 5,000 bootstrap resamples. Because the Negative PCD condition represents the theoretically critical case of upward social comparison—the scenario in which a viewer witnesses a peer being favored—we focused the mediation on this contrast. The model included Condition (dummy coded: Negative PCD = 1 vs. Control/Positive = 0) as the predictor (X), measured PCD score as the mediator (M), and Engagement as the outcome (Y), controlling for baseline preference. First, the manipulation significantly predicted the mediator (PCD score) (a=−2.32,p<.001). Second, the PCD score significantly predicted Engagement (*b* = 0.15, *p* < .001). The analysis of the indirect effect confirmed mediation: the indirect effect of witnessing peer interactions on engagement *through* PCD was significant (Effect = −0.35, *SE* = 0.09, 95% CI [−0.55, −0.19]). The direct effect remained significant but reduced ($c^{\prime }=-0.71,p<\mathrm{.001}$), indicating partial mediation. H2 is supported. Taken together, Study 1 indicates that PCD accounts for part of the association between witnessed peer interactions and engagement, independent of viewers’ initial preferences.

### Study 2: The moderating role of streamer type

Study 2 tested whether the nature of the streamer (Emotional vs. Utility) moderates the PCD effect across different content types (H3). A 3 (PCD Condition) × 2 (Streamer Type) ANOVA revealed a significant two-way interaction, $F(2,414)=8.65,p<\mathrm{.001},\eta_{p}^{2}=\mathrm{.04}$.

**Fig 2:**
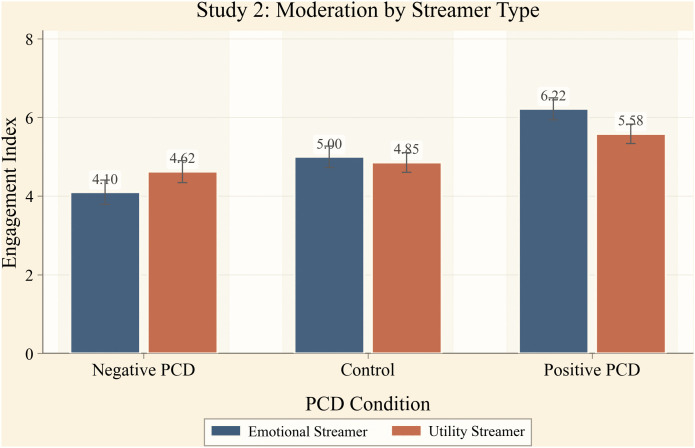
Study 2 results showing the PCD x Streamer Type interaction. The effect of PCD on engagement is significantly stronger for the emotional streamer than for the utility streamer.

We decomposed this interaction using simple effects analysis (see Fig 2). As illustrated in the figure, the effect of PCD was more pronounced in the Emotionally-Engaged context ($F(2,207)=52.54,p<\mathrm{.001},\eta_{p}^{2}=\mathrm{.34}$). The difference between Negative PCD (*M* = 4.10), Control (*M* = 5.00), and Positive PCD (*M* = 6.22) was substantial ($\Delta M_{Pos-Neg}=2.12,p<\mathrm{.001}$). In contrast, in the Utility-Focused context, while the main effect of PCD remained significant ($F(2,207)=14.37,p<\mathrm{.001},\eta_{p}^{2}=\mathrm{.12}$), the effect size was smaller. The difference between Negative PCD (*M* = 4.62), Control (*M* = 4.85), and Positive PCD (*M* = 5.58) was attenuated ($\Delta M_{Pos-Neg}=0.96$). These results support H3 and indicate that streamer type (emotional vs. utility) is an important contextual boundary condition of the PCD effect.

### Study 3: Moderation by viewer disposition

Study 3 examined the same boundary-condition question from the viewer’s perspective by testing whether the indirect effect of PCD is conditional on the viewer’s affective disposition (H4). Using PROCESS Model 7 (Moderated Mediation), we tested the interaction between Condition and Viewer Disposition (W) on the path to PCD (Path *a*). The interaction term was significant (*b* = 0.70, *SE* = 0.17, *p* < .001). The *Index of Moderated Mediation* was significant (Index = 0.11, 95% CI [0.05, 0.19]), confirming that the mediation strength varies by disposition.

**Fig 3:**
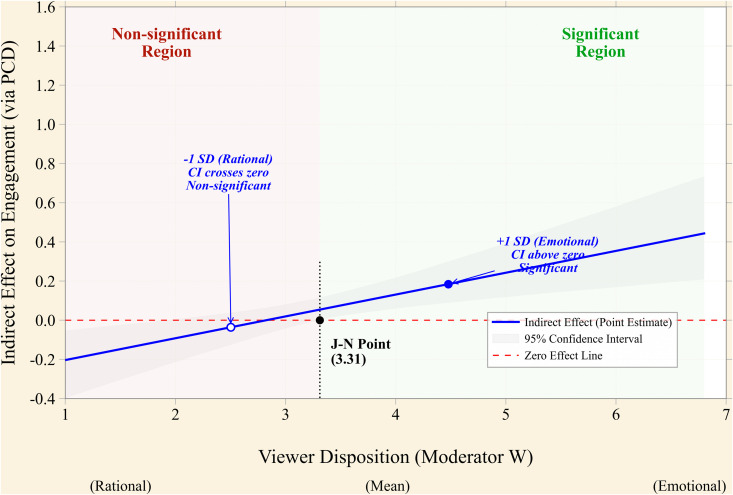
Johnson-Neyman floodlight analysis of the indirect effect of Condition on Engagement via PCD, moderated by Viewer Disposition (Study 3). The solid blue line represents the point estimate of the indirect effect, and the shaded area indicates the 95% bootstrap confidence interval. The indirect effect is significantly negative at low levels of viewer disposition (below 1.92), non-significant across the mid-range, and significantly positive once viewer disposition exceeds 3.31.

To probe this effect, we used the Johnson-Neyman (J-N) technique to identify the region of significance (see Fig 3). The floodlight analysis showed that the indirect effect of peer treatment on engagement via PCD was significantly negative for viewers scoring below 1.92, non-significant across the mid-range from 1.92 to 3.31, and significantly positive for participants scoring above 3.31 on the emotional disposition scale. These results support H4 and suggest that viewer disposition serves as an individual-level boundary condition alongside the contextual boundary established in Study 2.

### Study 4: The role of social identity

Study 4 tested whether the peer’s group identity moderates the impact of PCD (H5). A 2 (PCD: Negative vs. Positive) × 2 (Peer Identity: In-Group vs. Out-Group) ANOVA revealed a significant two-way interaction, $F(1,436)=6.21,p=\mathrm{.013},\eta_{p}^{2}=\mathrm{.01}$.

**Fig 4:**
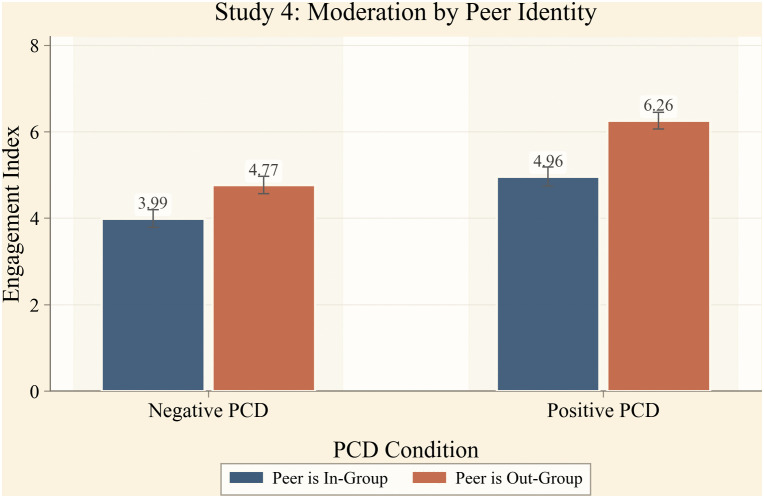
Study 4 results showing the PCD x Peer Identity interaction. Negative PCD (favoritism) is most damaging when the peer is in-group. Positive PCD (rejection) is most beneficial when the peer is out-group.

Simple effects analysis, visualized in Fig 4, clarified the mechanism:

- **Under Negative PCD (Favoritism):** Participants reacted more negatively when an *In-Group* peer was favored (*M* = 3.99) compared to an *Out-Group* peer (*M* = 4.77), |*t*|(218) = 5.33, *p* < .001, *d* = 0.72. This supports H5a and is consistent with an in-group favoritism threat interpretation.
- **Under Positive PCD (Rejection):** As shown in the right side of Fig 4, participants reported higher engagement when an *Out-Group* peer was rejected (*M* = 6.26) compared to an *In-Group* peer (*M* = 4.96), |*t*|(218) = 8.69, *p* < .001, *d* = 1.17. This supports H5b (Intergroup distinctiveness).

These findings support H5a and H5b, indicating that the social identity of the peer (in-group vs. out-group) is an important moderator of PCD effects, consistent with Social Identity Theory.

### Study 5: Public vs. private engagement

Study 5 examined the behavioral scope of PCD by distinguishing between public/social and private/financial engagement types (H6). A total of 330 participants were tested using a Multivariate Analysis of Variance (MANOVA) with two dependent variables: Public/Social Engagement and Private/Financial Engagement. The multivariate effect of PCD was significant, Wilks’ Λ=0.72,F(4,652)=29.47,p<.001.

Univariate follow-up tests revealed differential effects across engagement types:

- **Public/Social Engagement:** The effect of PCD was significant, $F(2,327)=64.10,p<\mathrm{.001},\eta_{p}^{2}=\mathrm{.28}$. Post-hoc tests showed a clear ordering between the three conditions ($M_{Pos}>M_{Con}>M_{Neg}$).
- **Private/Financial Engagement:** The effect was non-significant, $F(2,327)=1.78,p=\mathrm{.171},\eta_{p}^{2}=\mathrm{.01}$. Although the trend was consistent ($M_{Pos}=5.16$, $M_{Con}=4.93$, $M_{Neg}=4.90$), the manipulation did not significantly alter willingness to pay.

This supports H6 and indicates that PCD—as a social-comparative construct—primarily relates to public, reputation-based behaviors rather than transactional ones.

### Study 6: Downstream consequences on loyalty

Study 6 examined whether PCD relates to longer-term outcomes in streamer trustworthiness and viewer loyalty through perceived trust (H7). We tested a serial mediation model (PROCESS Model 6): Condition → PCD → Trustworthiness → Loyalty. The specific indirect effect was significant (Effect = 0.09, 95% CI [0.05, 0.16]). The specific path analysis revealed: 1. Condition significantly predicted PCD (*b* = 1.04, *p* < .001). 2. PCD significantly predicted Perceived Trustworthiness (*b* = 0.14, *p* < .001). 3. Perceived Trustworthiness significantly predicted Loyalty Intentions (*b* = 0.65, *p* < .001). These results suggest that single instances of PCD can function as diagnostic cues for streamer trustworthiness, with downstream implications for loyalty. Study 6 therefore extends the analysis beyond immediate engagement to longer-term relational outcomes.

## Discussion

Across six studies, we introduced, defined, and validated the Perceived Closeness Difference (PCD) as a critical construct for understanding viewer engagement. Taken together, the findings suggest a consistent pattern. Study 1 showed that witnessing differential treatment of a peer viewer was associated with PCD and with changes in engagement beyond viewers’ initial preference. Studies 2 and 3 identified boundary conditions, showing that this pattern was stronger for *emotionally-engaged streamers* and for more *emotionally-oriented viewers*. Study 4 showed that the *social identity* of the peer target (in-group vs. out-group) moderated the effect, consistent with Social Identity Theory. Study 5 narrowed the behavioral scope of the effect, showing stronger differences for *public, social* responses than for *private, financial* responses. Finally, Study 6 linked PCD-related events to *loyalty* through *streamer trustworthiness*.

This research makes several contributions to theory. First, we extend parasocial relationship theory [6,7] by moving beyond the dyad. The present findings provide empirical support for a one-sided to one-and-a-half-sided [11] or community-based [3] account of parasocial engagement. PCD offers one way to operationalize and measure social-comparative dynamics within this community, providing a mechanism for cross-viewer influence.

Second, we bridge media psychology and organizational behavior. By drawing a parallel between streamer-viewer and leader-member dynamics, we outline a *Viewer-Member Exchange* (VMX) differentiation perspective—a framework in which streamers, like organizational leaders, develop differentiated relationships with individual community members, and viewers, like employees, are sensitive to how their treatment compares to that of their peers. Our findings on PCD are broadly consistent with organizational behavior research on Relative LMX [17] and LMX differentiation [27]. Furthermore, the interactive experimental paradigm used here may also be useful for organizational behavior researchers examining LMX differentiation in virtual settings [15,33].

Third, we provide a new, dynamic, and ecologically valid paradigm for social comparison theory. We operationalize Negative PCD as an acute upward social comparison event and Positive PCD as a downward comparison event. Our findings—disengagement (for Negative PCD) and reinforced engagement (for Positive PCD)—represent the behavioral consequences of the self-esteem threats [13,24] and boosts [25] documented in social psychology.

Finally, the findings from Study 6 suggest that *parasocial fairness* may be an important antecedent of trust and loyalty. While trust is a well-established driver of loyalty in live streaming [4,34], an emerging but separate body of work has identified perceived fairness (interpersonal, distributive, etc.) as an antecedent to parasocial bond formation [20,35,36]. Our study experimentally *manipulates* this form of interpersonal fairness (via a salient PCD event) and shows that it is associated with perceived streamer trustworthiness, which in turn predicts loyalty.

### Comparison with existing literature

To situate our findings within the broader landscape of research, we briefly compare the present work with key prior studies across four dimensions.

First, regarding the *relationship model*, Kowert and Daniel [11] proposed the theoretical shift from “one-sided” to “one-and-a-half-sided” parasocial relationships, but their study was conceptual in nature. Our work provides an empirical examination of this triadic dynamic (Self–Streamer–Peer) by operationalizing PCD as a measurable construct and testing its association with engagement across multiple experiments.

Second, regarding *social comparison in digital environments*, prior studies such as Vogel et al. [13] and Liu et al. [26] investigated social comparison on static social media platforms (e.g., Facebook, Instagram) using correlational designs. Our study extends this literature to the *real-time, interactive* environment of live streaming with a *causal, experimental* approach. While Vogel et al. focused on self-esteem as an outcome of passive profile viewing, our PCD construct captures the immediate, vivid comparison that occurs when differential treatment is witnessed *as it happens*.

Third, regarding *LMX differentiation*, Lee et al. [17] and Choi et al. [19] demonstrated the detrimental effects of perceived LMX differences in workplace settings. Our study applies this organizational perspective to a consumer/media context, suggesting that related dynamics—sensitivity to differential treatment, justice perceptions, and behavioral adjustment—also operate in streamer–viewer communities. Notably, the effect sizes in our emotional streaming context ($\eta_{p}^{2}=\mathrm{.34}$) remain substantial and suggest that the public nature of live streaming may strengthen these dynamics.

Finally, regarding the *trust–loyalty pathway*, Kim et al. [4] and Tian et al. [34] established that trust in streamers predicts loyalty, identifying parasocial intimacy as the primary antecedent. Our Study 6 complements this by demonstrating that *fairness*—not just intimacy—is an independent and experimentally manipulable antecedent of trust. Where prior work modeled trust as arising from the quality of the viewer’s own relationship with the streamer, we show that trust can be undermined (or reinforced) by the streamer’s *observed behavior toward third parties*.

The findings offer practical implications for streamers, content creators, and platform managers. First, visible fairness appears to matter. Across studies, the *Negative PCD* (favoritism) condition was associated with the lowest engagement. This suggests that repeatedly validating one “top fan” at the expense of others may reduce engagement among viewers who witness that pattern. Second, **manage the community, not just the dyad**. Streamers must shift their perspective from managing thousands of individual viewer-streamer dyads to managing a *community*. This means ensuring that interactions, even positive ones, are distributed fairly and that all viewers feel a sense of belonging and equitable treatment. Third, we observe a possible engagement benefit of fair rejection. Witnessing a *negative* peer interaction (Positive PCD) was associated with higher engagement. This pattern is consistent with downward social comparison [25]. Streamers who enforce community rules (e.g., banning a “troll”) or reject a poor suggestion may, under some conditions, increase the engagement of other viewers who witness it by reinforcing a positive PCD and strengthening in-group/out-group boundaries [37].

This research is not without limitations. First, while our interactive experimental design enhanced experimental control and causal inference, it is still a lab setting. Future research should seek to replicate these findings in the field, perhaps by scraping chat logs and correlating gifting/subscription behaviors with public “shout-outs” from a streamer. Second, our studies examined a single, acute PCD event. Future longitudinal research should explore the effects of *chronic* or *consistent* PCD (e.g., the long-term impact on a viewer who is *always* ignored). Third, our exploration of social identity in Study 4 was a first step. This could be expanded to explore more complex inter-group dynamics, such as rival fan “cliques” within a single streamer’s channel [38]. Finally, while we propose a self-esteem-based mechanism, future work could explicitly measure mediating emotions like *envy* [26,39], which is a common outcome of upward social comparison.

## Conclusion

The dynamics of live streaming involve more than a series of one-to-one interactions. This research extends dyadic perspectives in media psychology by showing that a viewer’s engagement is also related to the witnessed treatment of others. We introduced and examined *Perceived Closeness Difference* (PCD) as a social-evaluative construct that captures this comparison process. Across six studies, viewers appeared to use streamer-peer interactions as cues to their own relative standing. When a peer was favored (Negative PCD), the focal viewer tended to withdraw; when a peer was rejected (Positive PCD), the focal viewer tended to report higher engagement.

Theoretically, this work connects parasocial interaction research with organizational behavior. By developing a Viewer-Member Exchange differentiation perspective, we provide empirical support for extending one-sided models toward a one-and-a-half-sided relationship account. The finding that PCD relates more strongly to public, reputation-based behaviors (commenting, sharing) than to private, transactional ones (gifting) further suggests that live streaming should be understood as a social as well as economic environment.

Practically, these findings suggest that visible favoritism may carry community-level costs. In the pursuit of monetizing “super fans,” streamers may unintentionally alienate other viewers through differential treatment. Sustainable community growth therefore requires attention not only to individual relationships, but also to relationship patterns within the channel. As digital interactions become increasingly immersive, understanding these triangular dynamics—Self, Streamer, and Other—may become increasingly important for explaining engagement in virtual spaces.

Beyond its academic and industry implications, this research also has broader societal relevance. First, PCD suggests that unfair treatment in live streams may pose a subtle threat to viewers’ *self-esteem and psychological well-being*, contributing to the growing body of evidence that digital social comparison processes have mental health consequences [13,24]. Awareness of PCD mechanisms can inform the design of healthier digital platforms that promote equitable and inclusive interactions. Second, our findings have implications for *platform governance and policy*: platforms may consider developing algorithmic tools or community guidelines that flag or mitigate visible favoritism, thereby reducing the social-comparative harm documented in our studies. Third, understanding PCD dynamics contributes to *digital literacy* efforts by equipping users—particularly younger audiences who constitute a large share of live-streaming consumers—with the knowledge to recognize and resist social comparison traps in interactive media environments. Finally, our results highlight an issue of *equity in the creator economy*: viewers may be disadvantaged by streamer behaviors that privilege a vocal minority, with implications for fairness in digital labor, consumption, and community participation.
