# Supplementary material for: Behind the screen: Unraveling the role of perceived closeness difference in shaping viewers’ engagement behaviors
Source: PLoS One. 2026 Jul 6;21(7):e0347751. doi: 10.1371/journal.pone.0347751 (PMC13336182; doi:10.1371/journal.pone.0347751)
Supplement: Attachment — Submitted filename: response_reviewer2.pdf [file pone.0347751.s002.pdf]

Response to Reviewer #2

Manuscript: Behind the Screen: Unraveling the Role of Perceived Closeness Difference in Shaping Viewers' Engagement Behaviors

March 20, 2026

Dear Reviewer #2,

Thank you very much for your thoughtful review and for your highly encouraging assessment of our manuscript. We are deeply grateful for the time you took to read our work and for your precise, insightful summary of our methodology and theoretical contributions.

Reviewer Comment: *Methodologically, the article is robust. The use of six experiments allows not only to establish causality, but also to identify boundary conditions; also they developed a simulator based on Python and HTML5 that mimics real platforms such as TikTok or Twitch, increasing the realism of the experience for the participant. As well, the study separates public from private engagement, allowing for a more nuanced understanding of how social status influences different types of user actions.*

Traditionally, parasocial interaction was studied as a one-to-one relationship. This research justifies the shift to a "one-and-a-half-sided" relationship model, which recognizes the presence of third parties in the content consumption process. It is pertinent the use of SCT to explain how differential treatment acts as a "relational thermostat" that triggers threats or boosts to the viewer's self-esteem.

In terms of results, the article reveals a counterintuitive benefit to "fair" rejection; for example, rejecting a bad suggestion can increase the engagement of other viewers by reinforcing positive social comparison. It suggests that streamers should stop viewing their channel as thousands of individual relationships and start managing it as a social ecology where every interaction is public and shared.

Response: We sincerely thank you for this incredibly positive and accurate synthesis of our core arguments. It is highly rewarding to see that our methodological choices—particularly the custom live-streaming simulator—and our theoretical shift toward a "one-and-a-half-sided" community ecology resonated so strongly with you.

While we are thrilled by your positive evaluation, we recognize that this study is only an initial step in unraveling the complexities of community dynamics in live streaming. Your comments have reinforced our commitment to this line of inquiry, and we aim to remain humble regarding the scope of our current findings.

As we look to the future, your summary inspires us to push this research forward in several directions to address the inevitable limitations of our current lab-based design. Specifically, our subsequent work will focus on:

1. **Field Validation:** Moving beyond the simulator to conduct field studies on actual platforms (e.g., scraping real-time chat logs to correlate gifting/subscription behaviors with streamer "shout-outs").

2. **Longitudinal Dynamics:** While our current study captures an acute, single instance of Perceived Closeness Difference (PCD), we plan to investigate the effects of *chronic* or consistent PCD (e.g., the long-term impact on a viewer who is repeatedly ignored).

3. **Complex Group Dynamics:** We hope to deepen our exploration of social identity by examining more complex inter-group dynamics, such as intense rivalries between different fan "cliques" within a single streamer's channel.

4. **Affective Mediators:** We plan to explicitly measure specific emotional mediators—such as envy or jealousy—which we suspect are the direct affective drivers bridging the cognitive PCD evaluation and the resulting behavioral engagement.

Once again, we wish to express our profound gratitude for your supportive review. Your endorsement gives us great confidence in the value of treating digital channels as interconnected social ecologies rather than merely collections of isolated dyads.

Sincerely,
Huichao Guo, on behalf of Runhua Huang
