# Supplementary material for: Behind the screen: Unraveling the role of perceived closeness difference in shaping viewers’ engagement behaviors
Source: PLoS One. 2026 Jul 6;21(7):e0347751. doi: 10.1371/journal.pone.0347751 (PMC13336182; doi:10.1371/journal.pone.0347751)
Supplement: Peer review history

# Peer review: Decision Letter 0

**DOI:** 10.1371/journal.pone.0347751.r001

**Authors:** José Luiz Vieira

1 Feb 2026

PONE-D-25-63717

Dear Dr. Guo,

Thank you for submitting your manuscript to PLOS ONE. After careful consideration, we feel that it has merit but does not fully meet PLOS ONE’s publication criteria as it currently stands. Therefore, we invite you to submit a revised version of the manuscript that addresses the points raised during the review process.

==============================

**ACADEMIC EDITOR:**

**Dear Dr. Guo,**

After a careful review of your manuscript, I believe that **minor revisions**  are required to address a few outstanding questions. Based on the **PLOS ONE**  editorial criteria, we recommend the following changes:

1. A brief review of past work should be included so as to make connect with the study which was carried.

2. At some places the proper flow is missing, for eg each study should clearly reflect to which extent the aim of study(1-4) was met.

3. A brief comparison of this work should also be added with the existing ones.

4. The conclusion should describe how this study is beneficial as a whole for the society.

Best regards

Jose luiz vieira

==============================

Please submit your revised manuscript by Mar 18 2026 11:59PM. If you will need more time than this to complete your revisions, please reply to this message or contact the journal office at plosone@plos.org. When you're ready to submit your revision, log on to https://www.editorialmanager.com/pone/ and select the 'Submissions Needing Revision' folder to locate your manuscript file.

Please include the following items when submitting your revised manuscript:

- A letter that responds to each point raised by the academic editor and reviewer(s). You should upload this letter as a separate file labeled 'Response to Reviewers'.
- A marked-up copy of your manuscript that highlights changes made to the original version. You should upload this as a separate file labeled 'Revised Manuscript with Track Changes'.
- An unmarked version of your revised paper without tracked changes. You should upload this as a separate file labeled 'Manuscript'.

-->If you would like to make changes to your financial disclosure, please include your updated statement in your cover letter. Guidelines for resubmitting your figure files are available below the reviewer comments at the end of this letter.

If applicable, we recommend that you deposit your laboratory protocols in protocols.io to enhance the reproducibility of your results. Protocols.io assigns your protocol its own identifier (DOI) so that it can be cited independently in the future. For instructions see: https://journals.plos.org/plosone/s/submission-guidelines#loc-laboratory-protocols. Additionally, PLOS ONE offers an option for publishing peer-reviewed Lab Protocol articles, which describe protocols hosted on protocols.io. Read more information on sharing protocols at https://plos.org/protocols?utm_medium=editorial-email&utm_source=authorletters&utm_campaign=protocols.

We look forward to receiving your revised manuscript.

Kind regards,

José Luiz Fernandes Vieira

Academic Editor

PLOS One

Journal Requirements:

When submitting your revision, we need you to address these additional requirements.

1. Please ensure that your manuscript meets PLOS ONE's style requirements, including those for file naming. The PLOS ONE style templates can be found at

https://journals.plos.org/plosone/s/file?id=wjVg/PLOSOne_formatting_sample_main_body.pdf and

https://journals.plos.org/plosone/s/file?id=ba62/PLOSOne_formatting_sample_title_authors_affiliations.pdf

2. Please note that PLOS One has specific guidelines on code sharing for submissions in which author-generated code underpins the findings in the manuscript. In these cases, we expect all author-generated code to be made available without restrictions upon publication of the work. Please review our guidelines at https://journals.plos.org/plosone/s/materials-and-software-sharing#loc-sharing-code and ensure that your code is shared in a way that follows best practice and facilitates reproducibility and reuse.

3.  Thank you for stating the following in the Acknowledgments Section of your manuscript:

“This study is supported by Zhejiang Business College, Hangzhou (KT25002497 and SZYYB202402) and the Soft Science Research Program of Zhejiang Province, Hangzhou (2025C35083).”

We note that you have provided additional information within the Acknowledgements Section that is not currently declared in your Funding Statement. Please note that funding information should not appear in the Acknowledgments section or other areas of your manuscript. We will only publish funding information present in the Funding Statement section of the online submission form.

Please remove any funding-related text from the manuscript and let us know how you would like to update your Funding Statement. Currently, your Funding Statement reads as follows:

“This study is supported by Zhejiang Business College, Hangzhou (KT25002497 and SZYYB202402) and the Soft Science Research Program of Zhejiang Province, Hangzhou (2025C35083).”

Please include your amended statements within your cover letter; we will change the online submission form on your behalf.

4. Thank you for stating the following financial disclosure:

“This study is supported by Zhejiang Business College, Hangzhou (KT25002497 and SZYYB202402) and the Soft Science Research Program of Zhejiang Province, Hangzhou (2025C35083).”

Please state what role the funders took in the study.  If the funders had no role, please state: "The funders had no role in study design, data collection and analysis, decision to publish, or preparation of the manuscript."

If this statement is not correct you must amend it as needed.

Please include this amended Role of Funder statement in your cover letter; we will change the online submission form on your behalf.

5. When completing the data availability statement of the submission form, you indicated that you will make your data available on acceptance. We strongly recommend all authors decide on a data sharing plan before acceptance, as the process can be lengthy and hold up publication timelines. Please note that, though access restrictions are acceptable now, your entire data will need to be made freely accessible if your manuscript is accepted for publication. This policy applies to all data except where public deposition would breach compliance with the protocol approved by your research ethics board. If you are unable to adhere to our open data policy, please kindly revise your statement to explain your reasoning and we will seek the editor's input on an exemption. Please be assured that, once you have provided your new statement, the assessment of your exemption will not hold up the peer review process.

6. We note that Figure 1 in your submission contain copyrighted images. All PLOS content is published under the Creative Commons Attribution License (CC BY 4.0), which means that the manuscript, images, and Supporting Information files will be freely available online, and any third party is permitted to access, download, copy, distribute, and use these materials in any way, even commercially, with proper attribution. For more information, see our copyright guidelines: http://journals.plos.org/plosone/s/licenses-and-copyright.

We require you to either (1) present written permission from the copyright holder to publish these figures specifically under the CC BY 4.0 license, or (2) remove the figures from your submission:

(1) You may seek permission from the original copyright holder of Figure 1 to publish the content specifically under the CC BY 4.0 license.

We recommend that you contact the original copyright holder with the Content Permission Form (http://journals.plos.org/plosone/s/file?id=7c09/content-permission-form.pdf) and the following text:

“I request permission for the open-access journal PLOS ONE to publish XXX under the Creative Commons Attribution License (CCAL) CC BY 4.0 (http://creativecommons.org/licenses/by/4.0/). Please be aware that this license allows unrestricted use and distribution, even commercially, by third parties. Please reply and provide explicit written permission to publish XXX under a CC BY license and complete the attached form.”

Please upload the completed Content Permission Form or other proof of granted permissions as an "Other" file with your submission.

In the figure caption of the copyrighted figure, please include the following text: “Reprinted from [ref] under a CC BY license, with permission from [name of publisher], original copyright [original copyright year].”

(2) If you are unable to obtain permission from the original copyright holder to publish these figures under the CC BY 4.0 license or if the copyright holder’s requirements are incompatible with the CC BY 4.0 license, please either i) remove the figure or ii) supply a replacement figure that complies with the CC BY 4.0 license. Please check copyright information on all replacement figures and update the figure caption with source information. If applicable, please specify in the figure caption text when a figure is similar but not identical to the original image and is therefore for illustrative purposes only.

6. If the reviewer comments include a recommendation to cite specific previously published works, please review and evaluate these publications to determine whether they are relevant and should be cited. There is no requirement to cite these works unless the editor has indicated otherwise.

7. Please review your reference list to ensure that it is complete and correct. If you have cited papers that have been retracted, please include the rationale for doing so in the manuscript text, or remove these references and replace them with relevant current references. Any changes to the reference list should be mentioned in the rebuttal letter that accompanies your revised manuscript. If you need to cite a retracted article, indicate the article’s retracted status in the References list and also include a citation and full reference for the retraction notice.

[Note: HTML markup is below. Please do not edit.]

Reviewers' comments:

Reviewer's Responses to Questions

**Comments to the Author**

1. Is the manuscript technically sound, and do the data support the conclusions?

The manuscript must describe a technically sound piece of scientific research with data that supports the conclusions. Experiments must have been conducted rigorously, with appropriate controls, replication, and sample sizes. The conclusions must be drawn appropriately based on the data presented.

Reviewer #1: Yes

Reviewer #2: Yes

**********

2. Has the statistical analysis been performed appropriately and rigorously?

Reviewer #1: Yes

Reviewer #2: Yes

**********

3. Have the authors made all data underlying the findings in their manuscript fully available?

The PLOS Data policy requires authors to make all data underlying the findings described in their manuscript fully available without restriction, with rare exception (please refer to the Data Availability Statement in the manuscript PDF file). The data should be provided as part of the manuscript or its supporting information, or deposited to a public repository. For example, in addition to summary statistics, the data points behind means, medians and variance measures should be available. If there are restrictions on publicly sharing data—e.g. participant privacy or use of data from a third party—those must be specified.

Reviewer #1: Yes

Reviewer #2: Yes

**********

4. Is the manuscript presented in an intelligible fashion and written in standard English?

PLOS ONE does not copyedit accepted manuscripts, so the language in submitted articles must be clear, correct, and unambiguous. Any typographical or grammatical errors should be corrected at revision, so please note any specific errors here.

Reviewer #1: Yes

Reviewer #2: Yes

**********

5. Review Comments to the Author

Please use the space provided to explain your answers to the questions above. You may also include additional comments for the author, including concerns about dual publication, research ethics, or publication ethics. (Please upload your review as an attachment if it exceeds 20,000 characters)

Reviewer #1: 1. A brief review of past work should be included so as to make connect with the study which was carried.

2. At some places the proper flow is missing, for eg each study should clearly reflect to which extent the aim of study(1-4) was met.

3. A brief comparison of this work should also be added with the existing ones.

4. The conclusion should describe how this study is beneficial as a whole for the society.

Reviewer #2: Methodologically, the article is robust. The use of six experiments allows not only to establish causality, but also to identify boundary conditions; also they developed a simulator based on Python and HTML5 that mimics real platforms such as TikTok or Twitch, increasing the realism of the experience for the participant.

As well, the study separates public from private engagement, allowing for a more nuanced understanding of how social status influences different types of user actions.

Traditionally, parasocial interaction was studied as a one-to-one relationship. This research justifies the shift to a “one-and-a-half-sided” relationship model, which recognizes the presence of third parties in the content consumption process. It is pertinent the use of SCT to explain how differential treatment acts as a “relational thermostat” that triggers threats or boosts to the viewer's self-esteem.

In terms of results, the article reveals a counterintuitive benefit to “fair” rejection; for example, rejecting a bad suggestion can increase the engagement of other viewers by reinforcing positive social comparison. It suggests that streamers should stop viewing their channel as thousands of individual relationships and start managing it as a social ecology where every interaction is public and shared.

**********

6. PLOS authors have the option to publish the peer review history of their article (what does this mean?). If published, this will include your full peer review and any attached files.

If you choose “no”, your identity will remain anonymous but your review may still be made public.

**Do you want your identity to be public for this peer review?**  For information about this choice, including consent withdrawal, please see our Privacy Policy.

Reviewer #1: **Yes:** Dr. Ankita Srivastava

Reviewer #2: No

**********

[NOTE: If reviewer comments were submitted as an attachment file, they will be attached to this email and accessible via the submission site. Please log into your account, locate the manuscript record, and check for the action link "View Attachments". If this link does not appear, there are no attachment files.]

To ensure your figures meet our technical requirements, please review our figure guidelines: https://journals.plos.org/plosone/s/figures

You may also use PLOS’s free figure tool, NAAS, to help you prepare publication quality figures: https://journals.plos.org/plosone/s/figures#loc-tools-for-figure-preparation.

NAAS will assess whether your figures meet our technical requirements by comparing each figure against our figure specifications.

# Author comment: Author response to Decision Letter 1

**DOI:** 10.1371/journal.pone.0347751.r002

27 Mar 2026

Response to Reviewer #1

Dear Reviewer #1,

Thank you sincerely for your careful reading of our manuscript and your constructive feedback. Your comments have been instrumental in improving the clarity, coherence, and scholarly rigor of our work. Below, we provide a point-by-point response to each of your comments, detailing the specific revisions we have made.

Reviewer Comment 1:

A brief review of past work should be included so as to make connect with the study which was carried.

Response:

We greatly appreciate this suggestion. We agree that a dedicated literature review is essential for establishing the theoretical foundation and connecting it to our empirical studies.

In the revised manuscript, we have added a new section titled “Literature Review” between the Introduction and the Materials and Methods sections. This section is organized into four subsections:

(a) Parasocial Interaction and Live Streaming Engagement

This subsection traces the evolution of Parasocial Interaction (PSI) theory from its origins in Horton and Wohl (1956) and the operationalization by Rubin et al. (1985), through recent applications in live streaming contexts (e.g., Lee et al., 2024; Yen et al., 2024; Huang, 2025). We highlight the shift toward community-level perspectives, particularly the “one-and-a-half-sided” model proposed by Kowert and Daniel (2021), and identify the gap our study addresses: the lack of empirical models quantifying how witnessed third-party interactions affect the focal viewer.

(b) Social Comparison Theory in Digital Environments

This subsection reviews Festinger's (1954) foundational theory and its extension by Buunk and Gibbons (2007), followed by key empirical findings on social comparison effects in digital media, including self-esteem impacts on social media (Vogel et al., 2014; Le Blanc-Brillon et al., 2025), downward comparison effects (Rüther et al., 2023), and social media envy (Liu et al., 2024). We identify the gap that existing research has focused on static platforms, not real-time interactive environments.

(c) Leader-Member Exchange (LMX) Differentiation

This subsection reviews the LMX differentiation literature, including the meta-analytic evidence (Martin et al., 2016), conceptual clarifications (Martin et al., 2018), relative LMX effects (Lee et al., 2019), and team-level consequences (Choi et al., 2020; Liden et al., 2006). We draw the novel parallel between leader-member and streamer-viewer dynamics.

(d) Hypotheses Development

We have added a dedicated subsection that explicitly states all seven hypotheses (H1-H7) with their theoretical grounding, providing a clear link between the reviewed literature and our empirical studies.

Additionally, we have added three foundational references to strengthen the review: Rubin et al. (1985) on the PSI scale, Buunk and Gibbons (2007) on social comparison orientation, and Liden et al. (2006) on LMX differentiation.

Changes Made:

A new “Literature Review” section with four subsections has been inserted between the Introduction and Materials and Methods. Three new references have been added to the bibliography.

Reviewer Comment 2:

At some places the proper flow is missing, for eg each study should clearly reflect to which extent the aim of study(1-4) was met.

Response:

Thank you for this important observation. We acknowledge that the original manuscript did not sufficiently connect each study's findings back to our overarching research aims, which may have disrupted the reader's ability to follow the logical progression of our research program.

In the revised manuscript, we have improved the flow by explicitly mapping each study to the newly formalized hypotheses (H1-H7) developed in the Literature Review section. We have added clear orienting and concluding sentences to the Results section of each study:

Study 1 now opens by stating it examined whether witnessed peer interactions influence focal viewer engagement beyond personal preference (H1) and whether PCD mediates this relationship (H2), and explicitly concludes that H2 is supported, indicating that PCD causally mediates the impact.

Study 2 opens by stating it tested whether the nature of the streamer (Emotional vs. Utility) moderates the PCD effect (H3), and concludes that the results support H3 and indicate that streamer type is an important contextual boundary condition.

Study 3 opens by testing whether the effect is conditional on the viewer's affective disposition (H4), and closes by noting these results support H4 and suggest that viewer disposition serves as an individual-level boundary condition.

Study 4 opens by testing whether the peer's group identity moderates the impact of PCD (H5), and clarifies the mechanism by concluding these findings support H5a and H5b.

Study 5 delineates the behavioral scope by distinguishing between public/social and private/financial engagement types (H6), concluding that PCD primarily relates to public, reputation-based behaviors.

Study 6 addresses long-term outcomes, opening by examining whether PCD relates to streamer trustworthiness and viewer loyalty through perceived trust (H7), and concludes that it extends the analysis beyond immediate engagement to longer-term relational outcomes.

By explicitly stating the hypothesis being tested at the outset of each study and confirming whether it was supported at the conclusion, we believe the reader can now easily track how each study sequentially builds on the previous one to fulfill our research aims.

Changes Made:

Explicit hypothesis-linking opening and concluding sentences have been highlighted in all six Study subsections in the Results section.

Reviewer Comment 3:

A brief comparison of this work should also be added with the existing ones.

Response:

We thank the reviewer for this suggestion. We agree that a systematic comparison with existing work strengthens the positioning of our contribution.

We have added a new subsection titled “Comparison with Existing Literature” in the Discussion section. This subsection compares our findings with key prior studies across four dimensions:

(a) Relationship model

We compare our empirical quantification of the triadic dynamic (Self-Streamer-Peer) with the conceptual proposal by Kowert and Daniel (2021), noting that our study is the first to operationalize and causally test the “one-and-a-half-sided” relationship model.

(b) Social comparison in digital environments

We compare our real-time, experimental approach with the correlational, static-platform studies by Vogel et al. (2014) and Liu et al. (2024), highlighting that our PCD construct captures the immediate, vivid comparison unique to live streaming.

(c) LMX differentiation

We compare our cross-application of organizational theory to the consumer/media context with the workplace-focused studies by Lee et al. (2019) and Choi et al. (2020), noting that our effect sizes in the emotional streaming context are comparable to or larger than those in organizational settings.

(d) Trust-loyalty pathway

We compare our finding that fairness (not just intimacy) drives trust with the PSI-to-trust models of Kim et al. (2025) and Tian et al. (2023), distinguishing our experimental manipulation of fairness from their correlational approaches.

Changes Made:

A new “Comparison with Existing Literature” subsection has been added to the Discussion section.

Reviewer Comment 4:

The conclusion should describe how this study is beneficial as a whole for the society.

Response:

We appreciate this valuable suggestion. We agree that articulating the broader societal implications of our research is important for demonstrating its relevance beyond academic and industry contexts.

We have added a substantial new paragraph at the end of the Conclusion section that addresses four dimensions of societal benefit:

(a) Digital well-being and mental health

We discuss how PCD reveals that unfair treatment in live streams can threaten viewers' self-esteem and psychological well-being, contributing to the growing evidence on social media's mental health impact. We argue that awareness of PCD mechanisms can inform the design of healthier digital platforms.

(b) Platform governance and policy

We note that our findings provide empirical grounds for platform policies and algorithmic tools that can flag or mitigate visible favoritism, reducing social-comparative harm.

(c) Digital literacy

We argue that understanding PCD dynamics can contribute to digital literacy education, equipping users, particularly younger audiences, with the knowledge to recognize and resist social comparison traps in interactive media.

(d) Equity in the creator economy

We highlight that the “silent majority” of viewers can be systematically disadvantaged by streamer behaviors that privilege a vocal minority, with implications for fairness in digital labor, consumption, and community participation.

Changes Made:

A new paragraph on broader societal implications has been added at the end of the Conclusion section.

We hope that these revisions adequately address all of your concerns. We are grateful for your constructive feedback, which has significantly strengthened the manuscript. We remain open to any further suggestions you may have.

Response to Reviewer #2

Dear Reviewer #2,

Thank you very much for your thoughtful review and for your highly encouraging assessment of our manuscript. We are deeply grateful for the time you took to read our work and for your precise, insightful summary of our methodology and theoretical contributions.

Reviewer Comment:

Methodologically, the article is robust. The use of six experiments allows not only to establish causality, but also to identify boundary conditions; also they developed a simulator based on Python and HTML5 that mimics real platforms such as TikTok or Twitch, increasing the realism of the experience for the participant. As well, the study separates public from private engagement, allowing for a more nuanced understanding of how social status influences different types of user actions.

Traditionally, parasocial interaction was studied as a one-to-one relationship. This research justifies the shift to a “one-and-a-half-sided” relationship model, which recognizes the presence of third parties in the content consumption process. It is pertinent the use of SCT to explain how differential treatment acts as a “relational thermostat” that triggers threats or boosts to the viewer's self-esteem.

In terms of results, the article reveals a counterintuitive benefit to “fair” rejection; for example, rejecting a bad suggestion can increase the engagement of other viewers by reinforcing positive social comparison. It suggests that streamers should stop viewing their channel as thousands of individual relationships and start managing it as a social ecology where every interaction is public and shared.

Response:

We sincerely thank you for this incredibly positive and accurate synthesis of our core arguments. It is highly rewarding to see that our methodological choices, particularly the custom live-streaming simulator, and our theoretical shift toward a “one-and-a-half-sided” community ecology resonated so strongly with you.

While we are thrilled by your positive evaluation, we recognize that this study is only an initial step in unraveling the complexities of community dynamics in live streaming. Your comments have reinforced our commitment to this line of inquiry, and we aim to remain humble regarding the scope of our current findings.

As we look to the future, your summary inspires us to push this research forward in several directions to address the inevitable limitations of our current lab-based design. Specifically, our subsequent work will focus on:

Field Validation: Moving beyond the simulator to conduct field studies on actual platforms (e.g., scraping real-time chat logs to correlate gifting/subscription behaviors with streamer “shout-outs”).

Longitudinal Dynamics: While our current study captures an acute, single instance of Perceived Closeness Difference (PCD), we plan to investigate the effects of chronic or consistent PCD (e.g., the long-term impact on a viewer who is repeatedly ignored).

Complex Group Dynamics: We hope to deepen our exploration of social identity by examining more complex inter-group dynamics, such as intense rivalries between different fan “cliques” within a single streamer’s channel.

Affective Mediators: We plan to explicitly measure specific emotional mediators, such as envy or jealousy, which we suspect are the direct affective drivers bridging the cognitive PCD evaluation and the resulting behavioral engagement.

Once again, we wish to express our profound gratitude for your supportive review. Your endorsement gives us great confidence in the value of treating digital channels as interconnected social ecologies rather than merely collections of isolated dyads.

Sincerely,

Huichao Guo, on behalf of Runhua Huang

Attachment Submitted filename: response_reviewer2.pdf

# Editor report: Decision Letter 1

**DOI:** 10.1371/journal.pone.0347751.r003

**Authors:** José Luiz Vieira

7 Apr 2026

Behind the Screen: Unraveling the Role of Perceived Closeness Difference in Shaping Viewers’ Engagement Behaviors

PONE-D-25-63717R1

Dear Dr. Guo

We’re pleased to inform you that your manuscript has been judged scientifically suitable for publication and will be formally accepted for publication once it meets all outstanding technical requirements.

Within one week, you’ll receive an e-mail detailing the required amendments. When these have been addressed, you’ll receive a formal acceptance letter and your manuscript will be scheduled for publication.

An invoice will be generated when your article is formally accepted. Please note, if your institution has a publishing partnership with PLOS and your article meets the relevant criteria, all or part of your publication costs will be covered. Please make sure your user information is up-to-date by logging into Editorial Manager at Editorial Manager® and clicking the ‘Update My Information' link at the top of the page. For questions related to billing, please contact billing support.

If your institution or institutions have a press office, please notify them about your upcoming paper to help maximize its impact. If they’ll be preparing press materials, please inform our press team as soon as possible -- no later than 48 hours after receiving the formal acceptance. Your manuscript will remain under strict press embargo until 2 pm Eastern Time on the date of publication. For more information, please contact onepress@plos.org.

Kind regards,

José Luiz Fernandes Vieira

Academic Editor

PLOS One

# Editor report: Acceptance letter

**DOI:** 10.1371/journal.pone.0347751.r004

**Authors:** José Luiz Vieira

PONE-D-25-63717R1

PLOS One

Dear Dr. Guo,

I'm pleased to inform you that your manuscript has been deemed suitable for publication in PLOS One. Congratulations! Your manuscript is now being handed over to our production team.

At this stage, our production department will prepare your paper for publication. This includes ensuring the following:

* All references, tables, and figures are properly cited

* All relevant supporting information is included in the manuscript submission,

* There are no issues that prevent the paper from being properly typeset

You will receive further instructions from the production team, including instructions on how to review your proof when it is ready. Please keep in mind that we are working through a large volume of accepted articles, so please give us a few days to review your paper and let you know the next and final steps.

Lastly, if your institution or institutions have a press office, please let them know about your upcoming paper now to help maximize its impact. If they'll be preparing press materials, please inform our press team within the next 48 hours. Your manuscript will remain under strict press embargo until 2 pm Eastern Time on the date of publication. For more information, please contact onepress@plos.org.

You will receive an invoice from PLOS for your publication fee after your manuscript has reached the completed accept phase. If you receive an email requesting payment before acceptance or for any other service, this may be a phishing scheme. Learn how to identify phishing emails and protect your accounts at https://explore.plos.org/phishing.

If we can help with anything else, please email us at customercare@plos.org.

Thank you for submitting your work to PLOS ONE and supporting open access.

Kind regards,

PLOS ONE Editorial Office Staff

on behalf of

Dr. José Luiz Fernandes Vieira

Academic Editor

PLOS One
